# Nutritional Approaches in Children with Overweight or Obesity and Hepatic Steatosis

**DOI:** 10.3390/nu15112435

**Published:** 2023-05-23

**Authors:** Chiara Spiezia, Claudia Di Rosa, Danilo Fintini, Pietro Ferrara, Laura De Gara, Yeganeh Manon Khazrai

**Affiliations:** 1 Research Unit of Food Science and Human Nutrition, Department of Science and Technology for Sustainable Development and One Health, Università Campus Bio-Medico di Roma, Via Alvaro del Portillo, 21-00128 Roma, Italy; chiara.spiezia@unicampus.it (C.S.); l.degara@unicampus.it (L.D.G.); m.khazrai@unicampus.it (Y.M.K.); 2 Endocrinology and Diabetology Unit, Bambino Gesù Children’s Hospital, IRCCS L.go S.Onofrio, 4-00165 Roma, Italy; danilo.fintini@opbg.net; 3 Operative Research Unit of Pediatrics, Department of Medicine and Surgery, Fondazione Policlinico Universitario Campus Bio-Medico, Via Alvaro del Portillo, 200-00128 Roma, Italy; p.ferrara@policlinicocampus.it; 4 Operative Research Unit of Nutrition and Prevention, Fondazione Policlinico Universitario Campus Bio-Medico, Via Alvaro del Portillo, 200-00128 Roma, Italy

**Keywords:** NAFLD, childhood and steatosis, childhood obesity

## Abstract

Childhood obesity is a global public health problem. Worldwide, 41 million children under 5 years and 340 million children and adolescents between 5 and 19 years are overweight. In addition, the recent COVID-19 epidemic has further amplified this social phenomenon. Obesity is a condition associated with various comorbidities, such as nonalcoholic fatty liver disease (NAFLD). The pathophysiology of NAFLD in obesity is intricate and involves the interaction and dysregulation of several mechanisms, such as insulin resistance, cytokine signaling, and alteration of the gut microbiota. NAFLD is defined as the presence of hepatic steatosis in more than 5% of hepatocytes, evaluated by histological analysis. It can evolve from hepatic steatosis to steatohepatitis, fibrosis, cirrhosis, hepatocellular carcinoma, and end-stage liver failure. Body weight reduction through lifestyle modification remains the first-line intervention for the management of pediatric NAFLD. Indeed, studies suggest that diets low in fat and sugar and conversely rich in dietary fibers promote the improvement of metabolic parameters. This review aims to evaluate the existing relationship between obesity and NAFLD in the pediatric population and to assess the dietary patterns and nutritional supplementations that can be recommended to prevent and manage obesity and its comorbidities.

## 1. Introduction

The spread of obesity is reaching increasingly alarming proportions, both among adults and children [1,2]. Childhood obesity has become a serious public health issue. According to the World Health Organization’s (WHO) latest estimates, 41 million children under 5 years and 340 million children and adolescents between the age of 5 and 19 are overweight and affected by obesity worldwide. This trend is expected to increase in the future [3]. Data published by the Organization for Economic Cooperation and Development (OECD) show that Italian children have the highest overweight rate in Europe, after Greece, and they occupy fourth place for childhood obesity in the world [4]. In addition, the recent COVID-19 epidemic has further amplified this social phenomenon. A new term, “covibesity”, has been coined to describe the exacerbation of obesity rates due to the lockdown imposed during the pandemic [5]. According to a study by the Center for Disease Control and Prevention (CDC) conducted on more than 432 thousand children and youth between 2 and 19 years of age, the rate of body mass index (BMI) has doubled compared to the pre-pandemic period, and in the 6–11 age group it increased by two and a half times [6]. Several comorbidities and an increased risk of mortality are associated with obesity [7,8]. With the epidemic increase in childhood obesity in recent years, non-alcoholic fatty liver disease (NAFLD) steatosis has become the most frequent cause of pediatric liver disease.

This review aims to evaluate the existing relationship between obesity and NAFLD in the pediatric population and to assess the dietary patterns and nutritional supplementations (vitamin E, vitamin D, omega 3, and probiotics) that can be adopted to prevent and manage childhood obesity and its comorbidities.

## 2. Search Strategy

We searched for human clinical studies published in English from January 2012 up to December 2022 in children (<18 years old) with obesity and NAFLD. The research was conducted through the PubMed database, with the following keywords: “NAFLD”; “childhood and steatosis”; and “childhood obesity”. Studies were firstly evaluated on the abstract and then included in the paper if they met our inclusion criteria. Then, a further search was conducted through citations of included articles to identify other suitable works.

## 3. Childhood Obesity

### 3.1. Definition

Childhood obesity is one of the most serious public health challenges affecting every country in the world. The WHO defines obesity as a clinical condition characterized by excessive body weight due to fatty tissue accumulation to an extent that adversely affects the health status. To define if a subject is underweight, normal weight, overweight, or affected by obesity, the BMI calculation is used. It derives from a mathematical ratio between weight (expressed in kilograms) and height (expressed in meters square) (kg/m^2^) [9]. Generally, in the growth age, the BMI is substituted by percentiles to represent the distribution of body weight, height, and BMI in the reference population. The diagnosis of excess of weight in children up to 24 months of age is based on the weight/length ratio using the WHO 2006 reference curves which consider overweight risk: >85th percentile; overweight: >97th percentile; and obesity: >99th percentile [10]. At older ages (2–5 years) BMI is used, according to WHO 2006 reference curves which consider overweight: >85th percentile and obesity: >97th percentile; while at even older ages (5–18 years) the WHO 2007 reference curves consider overweight: >85th percentile and obesity: >97th percentile [10]. Diagnostic criteria to summarize the classification of overweight and obesity in children are reported in Table 1.

To assess a child’s proper growth and weight status, it is necessary to take into consideration anthropometric parameters such as weight, length up to age 2, and height from then on [11].

The measurement of waist circumference is useful too, based on the strong correlation between the distribution of body fat and metabolic complications. Thus, it is a good practice to determine the ratio between waist circumference and height in overweight children older than 5 years old. A value higher than 0.5 irrespective of sex, age, BMI, and ethnic group is associated with increased cardiovascular risk factors [10]. In addition to the above-mentioned anthropometric parameters, cutaneous fold measurements may also be used to assess subcutaneous fat in children. Specifically, tricipital plica is indicative of overweight if it is higher than the 85th percentile and of obesity if it is higher than the 95th percentile [10].

### 3.2. Epidemiology and Comorbidities

The United Nations Sustainable Development Goals aim at eradicating hunger and all three forms of malnutrition (stunting, wasting, and overweight) everywhere in the world by 2030 [12]. Unfortunately, we are still far from a world without malnutrition. Although the prevalence of stunting has been declining globally since 2000, nearly 40 million children under the age of 5 are overweight, accounting for nearly 6% of this age group [13]. Among children and teenagers between 5 and 19 years, rates are significantly higher: more than 340 million are estimated to be overweight, nearly 18%. While overweight and obesity were once more prevalent in high-income countries, they are now also increasing in low- and middle-income countries, especially in urban settings. In Africa, the number of overweight children under 5 years old has increased by almost 24% from 2000. In 2019, in Asia, nearly 50% of all children under 5 years old were overweight or affected by obesity [14].

Data published in 2019 by the OECD “Organization for Economic Cooperation and Development” showed that in 2016 the rates of pre-obesity and obesity in children and adolescents (5–19 years old) in OECD countries were 18.7% and 9.9%, respectively. In particular, the United States had the highest prevalence of pre-obesity (20.4%) and obesity (21.4%) followed by New Zealand, Greece, and Italy [15].

Italy is a member of the WHO’s project called “Childhood Obesity Surveillance Initiative” (COSI), which collected data from 411 thousand children between 6 and 9 years old during the three-year period 2018–2020 [16]. The overall prevalence of excess of weight (including both overweight and obesity) in boys and girls between 7 and 9 years was 29%, with a higher rate among boys (31%) compared to girls (28%), while the overall prevalence of obesity was approximatively 12% (14% of boys and 10% of girls) [16].

Data from the Italian survey “Okkio alla salute”, published in 2019, showed that overweight children were 20.4% and children affected by obesity were 9.4%. This survey confirmed that boys have a slightly higher obesity rate than girls (9.9% vs. 8.8%, respectively). A geographical distinction was also observed; in fact, southern Italian regions showed higher rates of excess of weight in both genders than northern regions [17].

Childhood obesity is associated with numerous comorbidities, such as type 2 diabetes mellitus (T2DM), dyslipidemia, obstructive sleep apnea (OSA), and hepatic steatosis, which were formerly considered as “adult” diseases. The risk of developing these comorbidities is typically related to the severity of obesity [18].

## 4. Non-Alcoholic Fatty Liver Disease (NAFLD)

One of the repercussions of the epidemic spread of obesity is an increase in the prevalence of a condition known as “fatty liver” or hepatic steatosis. Currently, the term NAFLD seems to be not adequate for children because it cannot be associated with alcohol consumption, thus the new term “pediatric fatty liver disease (PeFLD)” seems to be more correct [19]. However, for simplicity, in the present review this condition is still called NAFLD.

It comprises a wide spectrum of liver diseases, ranging from triglyceride accumulation in the hepatocytes (simple hepatic steatosis or NAFLD) to various degrees of inflammation and fibrosis (non-alcoholic steatohepatitis, NASH) to cirrhosis [20].

Pediatric NAFLD is defined as chronic hepatic steatosis in children (<18 years old), not dependent on genetic or metabolic disorders, infection, steatogenic drug use, alcohol consumption, or malnutrition [21].

In both adults and children, NAFLD is frequently associated with all the manifestations related to insulin resistance [19], such as visceral adiposity, hypertension, dyslipidemia, and early atherosclerosis [22,23].

The prevalence of this condition in children is very heterogeneous and varies according to the degree of obesity, ranging from 9.6% in normal-weight children to 38% in those affected by obesity [24,25].

NAFLD is more prevalent in males than females, both before and during puberty [26]. The different distribution of the disease in the two genders is due to hormone production during puberty [24]. Additionally, hormones are involved in the development of NAFLD and in the progression to NASH. During puberty, boys with obesity show an increase in liver steatosis from 40% to 51%, while there is a decrease from 17% to 12% [26] due to the steady estrogen increase in females [27].

### 4.1. The Physiopathology of NAFLD

To simplify the pathogenesis of NAFLD, the multiple-hits hypothesis is used [28]. Indeed, to date, a number of multiple factors, including insulin resistance, nutritional factors, adipokines, gut microbiota, and genetic and epigenetic factors, are considered to interact, causing liver failure and the progression from NAFLD to NASH in genetically predisposed or high-risk individuals [29,30,31].

Several genes that encode proteins and regulate lipid metabolism are associated with pediatric NAFLD. These genes include Patatin-like phospholipase domain containing 3 (PNPLA3), glucokinase regulatory protein (GCKR), and transmembrane 6 superfamily member 2 (TM6SF2) [32,33], and are independent of insulin resistance [33]. Specifically, PNPLA3 encodes for adiponutrin and is associated with triglycerides accumulation in the liver [34]. Its expression is regulated by carbohydrate and lipid intake, but currently the mechanism by which PNPLA3 causes steatosis remains still unclear [34].

Hepatic steatosis is reversible, and in its pathogenesis four mechanisms are involved: (1) increased absorption of fatty acids by the liver; (2) reduced transport of fat as triglycerides in the very low-density lipoprotein (VLDL); (3) decreased β-oxidation of free fatty acids; and (4) increased de novo lipogenesis (DNL) in the liver [35].

It has been observed that the accumulation of triglycerides and insulin resistance are central features in the development of hepatic steatosis [28,33]. Subjects with obesity and NAFLD are less responsive to the action of insulin, and they exhibit increased lipolysis in subcutaneous and visceral fat resulting in increased free fatty acid (FAA) circulation [36]. Numerous FFAs (also assumed through the diet) reach the liver, stimulating hepatocytes to produce triglycerides. The FFAs undergo β-oxidation or esterification to triglycerides, with storage as lipid droplets or incorporation into very low-density lipoproteins (VLDLs). An imbalance, therefore, between synthesis, influx, oxidation, and excretion results in the accumulation of triglycerides typical of hepatic steatosis [37].

In addition, the hypertrophy and increased size of fat cells facilitates the infiltration of macrophages into the fat that produce pro-inflammatory cytokines/chemokines, such as Tumor Necrosis Factor-α (TNF-α), Interleukin-6 (IL-6), and Monocyte Chemoattractant Protein-1 (MCP-1) [36]. These mechanisms underlie insulin resistance, which promotes the release of FAAs from adipocytes, reducing their hepatic oxidation [36].

Oxidative stress, or “REDOX imbalance”, also plays an active role in the development of NAFLD and its progression into NASH [38]. It denotes the set of alterations that occur in tissues, cells, and biological macromolecules when exposed to an excess of oxidizing agents. The resultant metabolic alterations determine an accumulation of damaging macromolecules, inducing liver injury [39,40].

The liver is an organ involved in numerous activities, for instance it regulates micronutrient metabolism, the modification of which can lead to the onset of NAFLD [41]. Indeed, the liver, among its many functions, plays an important role in detoxification processes, although detoxification itself represents a form of oxidative stress on the organ. The liver filters the blood and detoxifies exo- or endotoxins that could potentially damage it [42].

Physiologically, to ensure the oxidation of toxins and facilitate their excretion, biotransformation processes produce ROS. However, an overload of toxins and a deficiency of antioxidants (necessary to counter and neutralize the production of radicals) results in tissue damage and inflammation [43].

In fact, in some clinical studies it is shown how the use of antioxidant molecules, such as vitamin E and hydroxytyrosol [44] and *n*-3 PUFA supplementation, reduces oxidative stress, the onset of NAFLD, and progression to hepatocellular carcinoma [45].

The adipose tissue normally secretes adipokines, specifically leptin and adiponectin [46]. Excessive adipose tissue results in an imbalance in adipokine production (leptin, resistin, and adiponectin), promoting the deposition of triglycerides in the liver [33]. In particular, an increased production of leptin and resistin and a decreased production of adiponectin have been seen [47]. Adiponectin mediates an insulin-sensitizing effect through binding AdipoR1 and AdipoR2 receptors [46]. Insulin resistance, dyslipidemia, and atherosclerosis also decrease adiponectin, while weight loss significantly increases its plasma levels. On the other hand, obesity and excess subcutaneous fat increase leptin levels. Leptin inhibits appetite, stimulates thermogenesis, enhances fatty acid oxidation, decreases glucose, and reduces body weight and fat [46]. People affected by obesity have higher circulating levels of leptin, but reduced hypothalamic sensitivity to its anorectic action, resulting in increased inflammation and fibrosis.

During puberty, the prevalence of hepatic steatosis is twice as high in males as in females with obesity. It depends on several factors: exogenous influences (diet) and levels of sex hormones (androgens and estrogens) and adipokines (leptin and adiponectin) [28]. Regarding sex hormones, estrogens are known to have antioxidant properties: they promote insulin sensitivity by reducing gluconeogenesis and glycogenolysis, and promote non-visceral adipose tissue distribution and the use of lipids as an energy substrate. During puberty, estrogen production increases markedly in girls and only minimally in boys [28].

Adipokines show a sex-dependent pattern too. In boys and girls with obesity, leptin is elevated throughout puberty with a tendency to increase in girls and decrease in boys, probably due to the effect of androgens. In contrast, adiponectin levels are similar in males and females at the beginning of puberty, but decrease significantly through puberty. In males, however, there is a more severe adiponectin reduction due to elevated androgen levels, which are inversely proportional to adiponectin concentrations. Hence, in males, lower levels of estrogen, higher levels of androgens, and lower levels of both leptin and adiponectin, in respect to female concentrations of these hormones, tend to comprehensively promote greater progression of NAFLD [28].

### 4.2. The Diagnosis of NAFLD in Children

Thus, as a preventive measure, children with obesity should undergo instrumental or laboratory screening to identify NAFLD [25]. As recommended by the North American Society of Pediatric Gastroenterology, Hepatology and Nutrition (NASPGHAN) guidelines, the best screening test for NAFLD in children is the alanine aminotransferase (ALT) assay. The interpretation should be based on sex-specific reference ranges (normal ALT < 26 U/L in boys and <22 U/L in girls) [48].

In addition, the finding of liver hyper-echogenicity (bright liver) on ultrasonography (US), with or without increased transaminases, raises suspicion of NAFLD [49].

It is also important to exclude other causes of hepatopathy (viral hepatitis, Wilson’s disease, autoimmune hepatitis, At-1 deficiency, etc.). In selected cases by the pediatric gastroenterologist/hepatologist, liver biopsy should be indicated, although it has its contraindications due to its invasiveness [50].

In addition, as evidenced by studies in children with histologically confirmed NAFLD diagnosis, the use of serum markers such as retinol-binding protein 4, cytokeratin 18, and hyaluronic acid, which is related to the degree of liver damage and fibrosis, is promising [51,52].

Non-invasive investigations (magnetic resonance, computed tomography, elastography, and ultrasound elastography) are also promising, but their use is not still recommended in children [53].

### 4.3. Intestinal Dysbiosis in NAFLD/NASH

There is evidence that NAFLD is influenced by the gut microbiota. To describe the link between the gut and the liver, the term “gut-liver axis” has been coined. Through the portal vein, the liver receives most of its blood supply from the intestine after nutrient digestion and absorption and is therefore one of the organs most exposed to potentially toxic factors. Thus, quantitative and qualitative variations in the microbiota composition can actively contribute to the pathogenesis of several liver diseases, including NAFLD, alcoholic steatohepatitis, and cirrhosis [54,55].

Indeed, patients with NAFLD exhibit small intestinal bacterial overgrowth (SIBO) and increased intestinal permeability that exposes the liver to toxic microbial metabolites [56,57]. Furthermore, when comparing subjects with obesity and NAFLD with normal-weight patients, intestinal dysbiosis is observed with a reduced number of Bacteroidetes and an increase in Firmicutes [58].

Del Chierico et al. evaluated gut microbiota changes in pediatric patients with NAFLD using targeted metagenomics and metabolomics [59]. Overall, patients with NAFLD had increased levels of *Bradirizobium*, *Anaerococcus*, *Peptoniphilus*, *Propionibacterium acnes*, *Dorea*, and *Ruminococcus* and reduced proportions of *Oscillospira and Rikenellaceae* compared to healthy controls.

In addition, a recent case–control study of 75 children aged 7–16 years evaluated the role of the gut microbiota in the development of NAFLD. Through 16S ribosomal RNA amplicon sequencing, the composition of the fecal microbiota was assessed [60]. It was found that children with NAFLD had a reduced presence of anti-inflammatory and probiotic bacteria (e.g., *Faecalibacterium*, *Akkermansia*, and *Bifidobacterium adolescentis*), while harmful bacteria (e.g., *Staphylococcaceae*) were numerous [60].

In children with NASH, an abundance of butyrate-producing bacteria, *Clostridia* and *Bacteroidia* (e.g., *Faecalibacterium*, *Roseburia_inulinivorans*, *Roseburia_intestinalis*, and *Coprococcus_comes*), has been seen. These bacteria are associated with glucose, lipid, and water electrolyte metabolism (e.g., glucose, triglycerides, cholesterol, inorganic salt, total body water, etc.) [60]. This could mean that NAFLD severity is associated with the metabolism dysregulation of these macro- and micronutrients.

In addition, several studies on fecal microbiota in children and adolescents with NAFLD have shown intestinal dysbiosis (characterized by a decrease in microbial diversity and an increase in the Firmicutes/Bacteroidetes ratio) [61,62]. In particular, there is an increased absorption of free fatty acids resulting in lipotoxicity and inflammation due to the alteration of intestinal microbiota and the change in the permeability of the small intestine [62,63].

The disruption of intracellular tight junctions causes an increased gut permeability and it determines the passage of lipopolysaccharides (LPSs) from the gut to the circulation [64]. LPSs, derived from Gram-negative bacteria, lead to liver damage as they activate Toll-like receptor 4 (TLR4), which triggers pro-inflammatory gene expression, inflammasome formation, and ROS generation through NADPH oxidase 2 [64,65,66].

The process that leads to the onset of steatosis with the passage of bacterial endotoxins to the liver via the portal vein is defined as “endotoxemia” [67].

Diet may alter the gut microbiota. An increased consumption of processed foods has caused a rise in the ingestion of fructose from 30% to 500% in the last 40 years [68]. Indeed, it has been seen that excessive fructose consumption increases inflammation, cellular stress, gut hepatic dysbiosis, and steatosis, leading to hepatic lipogenesis resulting in insulin resistance. Hepatic inflammation also increases due to fructose metabolism by fructokinase (KHK-C), leading to uric acid generation, mitochondrial dysfunction, and oxidative stress. In addition, high-fructose diets (HfruDs) or high-fat diets induce dysbiosis, which is accompanied by local and systemic inflammation and hepatic fat infiltration [69,70,71]. In fact, it has been seen that high-fat diets determined an increased circulating level of LPSs in both adults [72] and children with NAFLD [66]. On the contrary, it has been observed that both probiotics and high-fiber diets show a reduction in liver inflammation and fat accumulation [73,74]. Therefore, a potential therapeutic approach for the treatment of NAFLD could be the modulation of the gut microbiota to revert dysbiosis.

### 4.4. Nutritional Deficiencies in Children with NAFLD

Following a healthy, balanced diet is essential to receive an adequate amount of micronutrients, which can also influence the immune system [75]. Patients with NAFLD/NASH tend to have micronutrient deficiencies that worsen the pathogenesis of the disease, especially with regard to oxidative stress [76]. Although it is difficult to observe the direct effect of hypovitaminosis on NAFLD, both vitamin E and vitamin D, fat-soluble vitamins, are often low in patients with NAFLD, but also with cirrhosis and hepatocellular carcinoma.

Vitamin E is a free radical scavenger, so it plays a protective role against oxidative stress that can lead to the progression from NAFLD to NASH. Since vitamin D absorption depends on the gut’s ability to absorb dietary fat, individuals with fat malabsorption associated with diseases such as certain forms of liver disease, cystic fibrosis, celiac disease, and inflammatory bowel disease (Crohn’s disease and ulcerative colitis when the terminal ileum is involved) are more vulnerable to vitamin D deficiency. In addition, subjects with obesity (BMI ≥ 30) may have blood vitamin D (25-hydroxyvitamin D) values lower than normal-weight subjects because an excess of subcutaneous fat sequesters most of the vitamin and alters its release into the circulation [77]. Furthermore, low vitamin D levels have been shown to correlate with metabolic syndrome, insulin resistance, dyslipidaemia, and obesity, but also with hepatic steatosis and fibrosis in patients with NAFLD, but not with higher stages of fibrosis [76,78,79,80]. Generally, a serum concentration <20 ng/mL defines deficiency, between 21 and 30 ng/mL defines insufficiency, while >30 ng/mL defines sufficiency [81]. Yodoshi et al. observed that in 234 patients, liver fibrosis status was higher in children with NAFLD with insufficient, but not deficient, vitamin D than in those with sufficient vitamin D [81]. In the literature, there are contrasting results regarding the association between vitamin D levels and the risk of developing NAFLD [76]; a recent meta-analysis showed that there is an existing correlation between them. In this meta-analysis, children and adolescents with NAFLD had lower levels of Vitamin D than controls [78].

In fact, Nobili et al. demonstrated a negative correlation between vitamin D levels and the severity of NAFLD and fibrosis in 73 children with high aminotransferase levels and hyperechogenic liver on ultrasonography [82]. Additionally, Cho et al. showed that 3878 adolescents with NAFLD (in which 80% had ALT > 30 U/L) presented hypovitaminosis D (<20 ng/mL) [83].

In the literature, there are no studies regarding omega 3 deficiency in children with NAFLD. In general, omega 3 is important for an efficient insulin action. In fact, a decreased concentration of PUFAs could be associated with a reduced insulin sensitivity, especially in the muscle cell membranes [84].

## 5. Dietary Patterns and Nutritional Supplementation for Pediatric NAFLD

### 5.1. Dietary Patterns

From a therapeutic point of view, the primary treatment for pediatric NAFLD is lifestyle modification: improved eating habits and increased physical activity [21,85]. Gradual weight loss is recommended in order to avoid a progression from NAFLD to NASH that may present with a rapid loss of weight [85].

Some dietary interventions have been evaluated in the pediatric population with obesity, such as the control of portion sizes, the glycemic load reduction, low-carbohydrate diets, meal replacement and time-restricted feeding, as well as very-low-calorie diets and protein-sparing modified fasting for cases of more severe obesity [86,87,88,89,90].

Ramon-Krauel et al. compared, in a 6-month study, two different dietary patterns: a low-glycemic diet and a low-fat diet in 17 children with obesity and hepatic fat fraction (HFF) > 9%. Both nutritional protocols were effective in significantly reducing ALT and HFF, without differences between the two groups [91].

It is clear, however, that to manage the excess of body weight in children, as well as in adults, a dietary plan is effective if it can be sustained over a long period of time [92,93]. Thus, the goal is to find the best dietary pattern to prevent, mitigate, or revert NAFLD and its progression to steatohepatitis. Good candidates for treating NAFLD are dietary patterns that can improve insulin resistance, oxidative stress, or inflammation [92]. There is evidence in the literature that energy restriction alone is not sufficient to manage NAFLD [94] and that following a balanced diet with physical activity is essential [95,96]. Grønbæk et al. observed that moderate exercise for 1 h/daily and calorie restriction in 117 children with obesity determined an average weight loss of 7.1 kg and a marked reduction in liver steatosis, insulin resistance, and transaminases [97].

According to the international guidelines, NAFLD management requires a reduced intake of calories, fats (especially saturated fatty acids and trans fatty acids), and fructose and, conversely, an increased intake of proteins (especially from white and lean meats or fish), dietary fibers, and *n*-3 polyunsaturated fatty acids (PUFAs) [98].

At present, although the type of dietary pattern recommended in cases of NAFLD has not yet been defined, some studies suggest that a “diet low in free sugars” (those sugars added to foods and beverages and occurring naturally in fruit juices) [85,99] and a “Mediterranean diet” may have additional benefits in the treatment of steatosis, independently of weight loss [100].

The current WHO guidelines suggest that the daily intake of free sugars should be limited to less than 10% for all people and less than 5% in specific circumstances [101].

Several recent studies suggest that a high intake of added sugars (es. high-fructose corn syrup, HFCS, and sucrose), included in the preparation of food and beverages, plays a role in the onset of NAFLD because they contribute to increasing glucose and fructose intake in the diet. Children tend to consume added sugars through sugar-sweetened beverages (SSBs) [102].

Sullivan et al. demonstrated that children with NAFLD are able to absorb and metabolize fructose more efficiently than lean subjects [103]. Schwimmer et al. evaluated the effect of a “diet low in free sugars” on 40 male adolescents aged 11–16 with histologically diagnosed NAFLD and evidence of active disease (hepatic steatosis > 10% and ALT ≥ 45 U/L) [99]. The intervention diet in this study provided a free-sugar intake of less than 3% of daily calories and, after 8 weeks, subjects who had followed this diet achieved a greater reduction in liver steatosis from 25% to 17% compared to those who had followed the habitual diet [99].

Mager et al. conducted a study on 7- to 18-year-old subjects with NAFLD and healthy controls, evaluating the effects of a modest reduction in fructose intake (total/free and HFCS) and of load and glycemic index on improving plasma markers of hepatic dysfunction and cardiometabolic risk [104]. Specifically, they observed significant reductions in the systolic blood pressure (SBP), body fat percentage (BF), and plasma concentrations of ALT), Apo-B100, and HOMA-IR both at 3- and 6-month visits [104]. Additionally, Jin et al. showed that reducing fructose intake in Hispanic American adolescents with NAFLD improved cardiometabolic parameters such as insulin sensitivity and h-CRP and LDL oxidation [105].

In another study, Schwarz et al. evaluated for 9 days the effect of a reduced fructose consumption in 41 nondiabetic Latino and African American children with obesity and metabolic syndrome identified as habitual high sugar consumers (>15% sugar, >5% fructose daily) regardless of calories consumed [106]. On day 10, the fructose restriction led to reductions in liver fat, visceral fat (VAT), and de novo lipogenesis (DNL) and improved insulin kinetics [106].

The Mediterranean diet (MD) was first coined by Dr. Ancel Keys and in 2010 it became an intangible heritage of humanity by UNESCO. This nutritional pattern has been and currently is the meeting point between cultures and populations around the Mediterranean basin [107]. This nutritional pattern is one of the healthiest and most balanced dietary patterns and involves the consumption of all foods, without any exclusions, with a high intake of vegetables, legumes, fresh fruits, nuts, olive oil, and cereals (preferably whole grains); and a moderate consumption of fish, dairy products (especially fresh cheese and yogurt), meat, and occasionally sweets [108]. It is precisely the synergistic combinations of nutrients characterized by the protective substances they contain with the right water intake and the practice of physical activity that make the MD unique and beneficial to physical and mental health [76,109]. This diet is able to positively modulate all conditions associated with steatosis, including visceral obesity, insulin resistance, dyslipidemia, and chronic inflammation [110,111,112].

In fact, Della Corte et al. evaluated the impact of the Mediterranean diet on children with obesity and NAFLD. Specifically, 243 patients with obesity were enrolled and they underwent abdominal ultrasonography and laboratory analysis; a subgroup of 100 children also underwent biopsy [113]. A clinical questionnaire, the Mediterranean Diet Quality Index for children and adolescents (KIDMED), was used to assess dietary adherence. Patients with a poor Mediterranean diet adherence had higher levels of insulin concentration during the oral glucose tolerance test (both at baseline and after 120 min) and higher values of HOMA-IR and HOMA-β (expression of IR and deterioration of insulin sensitivity) [113]. In addition, patients with unhealthy eating habits had elevated C-reactive protein (CRP) values, indicating an increased inflammatory status. In addition, the prevalence of low KIDMED score was significantly higher in patients with nonalcoholic steatohepatitis compared to the other groups; a poor adherence to the MD correlated to liver damage, a NAFLD activity score > 5, and grade 2 fibrosis [113].

### 5.2. Nutritional Supplementation

Although in the literature there is a lack of trials in the last 10 years on the use of supplements in children with NAFLD; various trials based on the use of antioxidants (particularly vitamin E), vitamin D, docosahexaenoic acid (omega-3), and probiotics have been tested with different results on NAFLD or NASH in children.

#### 5.2.1. Antioxidants

Oxidative stress plays a crucial role in the progression from NAFLD to NASH, and consequently the use of antioxidant molecules that can modulate stress is being evaluated. Alpha-tocopherol (vitamin E) is the most widely studied antioxidant in cases of liver disease in the pediatric age group [44,114] because it is the most effective in inhibiting lipid oxidation [114]. It has beneficial roles in liver disease, in fact it plays anti-inflammatory, antioxidant, and antiapoptotic functions [115]:

- The anti-inflammatory response is promoted by the vitamin’s action in reducing the expression of cytokines such as (TNF-α), interleukin (IL)-1, IL-2, IL-4, IL-6, and IL-8, and in increasing the levels of adiponectin, which suppresses hepatic fatty acid synthesis, reduces liver fibrosis, and prevents cirrhosis [116,117,118].
- The antioxidant effect is enhanced by an increased production of superoxide dismutase (SOD) and acts as a scavenger of hydroxyl, peroxyl, and superoxide radicals, protecting against lipid and low-density lipoprotein (LDL) peroxidation. Thus, increased consumption of vitamin E limits the increased oxidative stress typical of patients with NAFLD. In addition, vitamin E also stimulates the action of antioxidant enzymes, including catalase and glutathione peroxidase [117,119].
- The antiapoptotic effect is due to increased levels of the antiapoptotic protein BCL-2 and decreased levels of the proapoptotic proteins BCL-2 associated-X (BAX) and p53. Furthermore, vitamin E limits the activity of caspase-9 and cytochrome C, which regulate mitochondrial apoptosis, and caspases-8 and 3, involved in the apoptotic pathway [120].

Vitamin E has been suggested to manage liver disease alone or in combination, but a limitation of its use is its bioavailability when administered with oral preparations, the dosage, and the lack of long-term studies [44].

Ghergherehchi et al. conducted a double-blind placebo study on 33 children with obesity and NAFDL and allocated them to either a lifestyle modification + vitamin E (400 IU) group or a lifestyle modification + placebo group for 6 months. Both groups followed a hypocaloric diet and 2 h of physical activity every day. In both groups, significant changes in BMI, ALT, AST triglycerides, and total and LDL cholesterol were observed and the mean difference between the two groups were not significant, except for LDL cholesterol. Thus, they declared that vitamin E supplementation was not more efficient than placebo in improving steatosis in children with obesity and NAFLD [121].

D’adamo et al. conducted a 6-month study on 42 prepubertal children with obesity and NAFLD that were equally allocated to either a vitamin E supplementation intervention group (group 1) and a control group (group 2). Both groups received nutritional and physical activity recommendations and only group 1 also received 600 mg/day of vitamin E. The results showed a significant reduction in BMI, waist circumference, and fasting glucose in both groups compared to baseline. Only in group 1 were a significant decrease in HOMA-IR, insulin, total cholesterol, LDL-cholesterol, and ALT and an increase in HDL cholesterol observed. Obviously, only in the treated group, a significant increase in the concentration of vitamin E levels (es-RAGE) and a decrease in the concentration of the marker of oxidative stress (PGF-2α) were assessed. Moreover, variations in vitamin E were associated with changes in ALT, HOMA IR, and total cholesterol independently of BMI reduction. Thus, vitamin E has determined a reduced level of oxidative stress and of LDL cholesterol, and an improvement in insulin and lipid profile [122]. The reduction in ALT levels has been observed also by Sarkhy et al. in its systematic review and meta-analysis [123]. Shiasi et al. compared vitamin E supplementation (800 IU/day or 400 IU/day) to metformin in 119 children with obesity and NAFLD for 2 months and found that there were no statistical differences on ultrasonography after both treatments [124]. To date, in the literature there is not a clear consensus on the use of vitamin E alone in children with NAFLD, thus further studies are needed [93].

Vitamin E supplementation has also been evaluated in addition to hydroxytyrosol (HXT) or to ursodeoxycholic acid (UDCA). The first one is a phenol found in extra virgin oil and, in addition to vitamin E, it has been seen to improve oxidative stress, insulin resistance, and steatosis in children [124,125]. Nobili et al. conducted a study evaluating the effects of vitamin E + HXT on 80 children and pre-adolescents with biopsy-proven NAFLD [44]. In total, 40 subjects received 7.5 mg of HXT and 10 mg of vitamin E (treatment arm), while the others received 240 mg medium-chain triglycerides (MCTs) as a placebo (placebo arm) for 4 months. All patients followed a low-calorie diet and regular physical activity. During the protocol, 10 children dropped out, thus 70 children concluded the study. In the treatment arm, a significant reduction in triglycerides and insulin levels and an improvement in HOMA-IR were observed. To evaluate the antioxidant activity, glutathione (GSH) and the ratio between GSH and oxidized glutathione (GSSG) were assessed. In the treatment arm, these two markers significantly increased, while in the placebo arm they decreased. Moreover, the stage of NAFLD was also assessed. After 4 months, the number of children affected by severe steatosis significantly decreased while those affected by mild steatosis increased. The improvement of steatosis positively correlated to HXT [44].

Two years later, the same group of authors published an article on the same population evaluating the effects of HXT+VitE on systemic inflammation [106]. At the end of the treatment, levels of IL-1β and TNF-α decreased in both groups, while IL-6 decreased and IL-10 increased significantly only in the HXT+VitE group. Moreover, children in the treatment group showed a significant reduction in two oxidative stress parameters, 4-hydroxynonenal (4-HNE) and 8-hydroxy-2′-deoxyguanosine (8-OhdG), correlated to an improvement in triglyceride serum levels. The results showed that there was an existing negative correlation between the decrease in 8-OhdG with both steatosis and IL-10 levels [126].

Regarding the role of the ursodeoxycholic acid (UDCA), it has been seen that it is able to antagonize the progression of NAFLD/NASH [127] through the protection of hepatocytes from mitochondrial damage mediated by bile salts, the antiapoptotic signaling pathway, and immunomodulatory function [128]. Cho et al. evaluated the combined effect of UDCA (5–10 mg/day) and vitamin E (800 IU/day) on 20 children aged 7–14 years with obesity and NAFLD. As the first line of intervention, patients were advised to lose weight through lifestyle modification. Ursodeoxycholic acid (UDCA) and vitamin E therapy was performed in children whose liver function did not improve or whose weight reduction was not successful after lifestyle advice. Vitamin E and UCDA supplementation, associated with a significant BMI reduction, determined statistically significant improvements in AST, ALT, AST/ALT ratio, alkaline phosphatase, total bilirubin levels, and γ-GT at follow up, compared to the baseline. ALT levels decreased from an average of 151.2 IU/L to 54.5 IU/L, and AST levels normalized from 75.8 IU/L to 32.3 IU/L. In contrast, drug treatment alone, without BMI reduction, did not improve NAFLD parameters [129].

Table 2 shows all included studies on vitamin E supplementation in children with obesity and NAFLD.

#### 5.2.2. Vitamin D

Vitamin D is a fat-soluble hormone and it is synthetized in the skin by solar exposure and it is converted to a biologically active form firstly in the liver and then in kidneys.

Thus, hepatic dysregulation may also be involved in the impaired vitamin D metabolism. It regulates bone metabolism and calcium homeostasis, but it also has a role in hepatic fibrogenesis [76,78].

Currently, there are no FDA-approved drugs for the treatment of NAFLD. However, vitamin D, because of its good insulin-sensitizing and anti-inflammatory properties, could be a good treatment option for patients with NAFLD [130].

The only study that evaluated the effects of vitamin D supplementation in children was the one of El Amrousy et al. They conducted a 6-month randomized controlled clinical trial on 100 children with biopsy-proven NAFLD to assess the effectiveness of vitamin D supplementation. Patients were randomly assigned into two groups: a treatment group (2000 IU/day of vitamin D) and a placebo group. They evaluated anthropometric parameters, vitamin D and serum calcium levels, AST, ALT, total cholesterol, triglycerides, LDL and HDL cholesterol, fasting glycemia and insulinemia, and HOMA-IR. At the end of the study, significant improvements in hepatic steatosis and lobular inflammation by liver biopsy and a decrease in AST, ALT, triglycerides, LDL, fasting insulin, and glycemia and HOMA-IR were observed. Moreover, vitamin D levels and HDL cholesterol significantly increased in the treated group. This study suggests that vitamin D supplementation may be useful for children with NAFLD [131].

Table 3 shows all included studies on vitamin D supplementation in children with obesity and NAFLD.

#### 5.2.3. Polyunsaturated Fatty Acids (PUFAs)

Polyunsaturated fatty acids (PUFAs) comprise the ω-3 series: α-linolenic acid (18:3; ALA), eicosapentaenoic acid (20:5; EPA), and docosahexaenoic acid (22:6; DHA) and the ω-6 series: linoleic acid (18:2; LA), g-linolenic acid (18:3), and arachidonic acid (20:4 ARA). They are essential, in fact human cells are able to synthetize them in small amounts, the majority must be provided by an adequate nutritional intake [45]. The ω-3 series is present in fish (oily fish, salmon, cod, and trout), leafy vegetables, and nuts, while the ω-6 series in vegetable oils (corn, sunflower, soybean, and grape seed) and in nuts such as almonds, pistachios, hazelnuts, and peanuts. EPA and DHA have been associated with the prevention of rheumatoid arthritis, obesity, type 2 diabetes, cardiovascular and neurodegenerative diseases, asthma, irritable bowel syndrome, cancer, and kidney diseases [132]. They are also involved in ameliorating hepatic steatosis, reducing hepatic de novo lipogenesis, and increasing fatty acid β-oxidation [133,134,135].

Pacifico et al., in a double-blind, parallel-group, placebo-controlled randomized trial, evaluated the effect of a six-month DHA treatment in the pediatric population compared to a placebo group in reducing hepatic and visceral fat and associated cardiovascular disease (CVD) risk factors [136]. Children younger than 18 years old, with a BMI > 85th percentile, persistently elevated aminotransferase levels, evidence of hepatic steatosis on Magnetic Resonance Imaging (MRI), and liver biopsy consistent with NAFLD were enrolled and allocated either to a DHA or a placebo group. After 6 months, a reduction of 53.4% of liver fat was observed in the DHA group compared to 22.6% in the placebo group [136]. Visceral adipose tissue (VAT) and epicardial adipose tissue (EAT) were also reduced by 7.8% and 14.2%, respectively, in the DHA group, compared to 2.2% and 1.7% in the placebo group. Additionally, fasting insulin and triglycerides decreased significantly in the DHA-treated group. Thus, DHA supplementation in children and adolescents with NAFLD could be considered as a therapeutic option in the treatment of this condition [136].

Janczyk et al. observed, in a 6-month randomized multicenter double-blind placebo-controlled trial, the effect of omega 3 (450–1300 mg depending on body weight, EPA+DHA in 3:2 proportion) supplementation compared to placebo (sunflower oil in the same dose) in 76 children aged 6–19 with NAFLD. All patients were invited to follow a hypocaloric diet and to increase physical activity. ALT and GGT significantly decreased but no changes were observed in ALT levels compared to baseline [137].

In another study, DHA supplementation (250 mg/day and 500 mg/day) in 60 children with NAFLD allocated to three groups for 1 year reduced both triglyceride and ALT levels and improved steatosis compared to the placebo group. The different dosage was equally effective in reducing liver fat content [138].

Spahis et al. conducted a 6-month double-blind, one-way, crossover randomized trial in 2015 on 30 children aged 8–18 years with obesity and NAFLD. They were randomly assigned to two groups. The first phase of the study consisted of administering an omega 3 supplementation (2 g of fish oil/daily providing 1.2 g of omega 3) to a group and a placebo (sunflower oil) to the other group for 3 months, while in the second phase both groups received the omega 3 supplementation for the last 3 months. The supplementation was provided by four capsules containing 500 mg of fish oil (300 mg of omega 3 with 3.75 U vitamin E), while the placebo was composed of 500 mg of sunflower oil with 3.75 U vitamin E. Only 21 out of 30 subjects completed the study. NAFLD children presented higher values of ALT, fasting insulin levels, HOMA-IR index, C-reactive protein, TG levels, and lower levels of HDL cholesterol at baseline compared to 33 healthy children. The 21 NAFLD children showed an increase in the proportion of EPA and DHA at the expense of *n*-6 FAs after the treatment [139].

Some years later, the same group evaluated, on the same subjects (*n* = 20), the severity of disease by assessing transaminase levels, liver ultrasound, NAFLD activity score, and fatty liver index (FLI) across two groups: 9 with mNAFLD (moderate fatty liver) and 11 with sNAFLD (severe fatty liver) [45]. The mNAFLD group had normal blood levels of transaminases, steatosis score of 1–2 (according to the NAS), and FLI < 30, while sNAFLD subjects had twice the normal levels of transaminases, steatosis score ≥ 5, and FLI ≥ 60. Boys in the sNAFLD group were also more insulin resistant, hypertensive, and characterized by elevated lipid indicators of cardiovascular disease risk compared to the mNAFLD group. The sNAFLD group consumed 2 g of *n*-3 PUFAs for 6 months. At the end of the *n*-3 PUFA treatment, there was a significant increase in EPA and DHA concentrations in red blood cells and a reduction in FLI, ALT, and ALT/AST ratio, with signs of improvement of hepatic steatosis. In addition, an improvement was observed in both carotid intima thickness and lipid profile with reduction in TG, LDL-C, and Apo B, as well as Apo B/AI, total cholesterol/HDL-C, LDL-C/HDL-C, and Apo B/TG ratios, without improvement in HDL-C, Apo A-I, and Apo family members. In treated patients, reductions in serum oxLDL, leptin, and acyl ghrelin, and an increase in adiponectin (signs of improvement in oxidative stress and inflammation associated with the disease) were also detected [45]. Thus, the administration of *n*-3 PUFA in patients with these metabolic abnormalities is useful not only in reducing NAFLD, but also in preventing serious complications such as diabetes and cardiovascular disease [45].

One hundred and eight children (9–17 years old) with NAFLD were included in the double-blind treatment of Boyraz et al. and they were randomly assigned to either a PUFA group (diet + 1000 mg dose of PUFA) or a placebo group (diet + placebo) for 12 months + a dietary lifestyle intervention. The PUFA intervention determined higher HDL levels and lower Triglycerides, insulin, glycemia, and HOMA-IR [140].

Regarding omega 3 consumption, the studies’ results provide a strong recommendation that children with NAFLD should consume the appropriate quantity of fish per week or they have to be supplemented with omega-3 [93]. However, regarding supplementation, there are not many long-term studies [141].

Table 4 shows all included studies on Vitamin D supplementation in children with obesity and NAFLD.

#### 5.2.4. PUFAs + Vitamin D

Only one study in the literature compared DHA + vitamin D in 41 children (4–16 years) with NAFLD and vitamin D deficiency (<20 ng/dL). The children were randomized to the treatment arm or the placebo arm. Treatment consisted of a mixture of vitamin D + DHA orally once daily versus placebo for 24 weeks. For the dosage of DHA, the aforementioned study by Nobili et al. was taken into account, while for that of vitamin D, the American Academy of Pediatrics’ recommendation of administering between 600 and 1000 IU/day in adolescents at risk of vitamin D deficiency was used. Treatment improved insulin resistance, lipid profile, and ALT compared to placebo, and biopsies showed an improvement in liver damage (NAFLD activity score) [142].

Table 5 shows all included studies on PUFA + vitamin D supplementation in children with obesity and NAFLD.

#### 5.2.5. Probiotics

Probiotics are nonpathogenic living micro-organisms which may affect the health of the host by regulating the intestinal microbiota, producing antibacterial substances, improving epithelial barrier function, and reducing intestinal inflammation [143,144,145,146,147,148].

Although the molecular mechanism of probiotics is still not completely understood, it is clear that they can be used in the treatment of NAFLD as they modulate the gut microbiota. They also improve intestinal mucosal permeability and inhibit the inflammatory response [149].

Certainly, a better knowledge about the various bacterial species that populate the gut of individuals with NAFLD warrants a selection of appropriate probiotics to improve liver status. Commercially available probiotics are Bifidobacterium and Lactobacillus. Furthermore, it would appear that Bifidobacteria play an active role in the management of NAFLD and obesity [150]. Thus, their potential future use as probiotics in the treatment of the disease has to be further investigated. There are several studies in the literature that have investigated the use of probiotics in NAFLD, as they may be helpful in reducing the risk of insulin resistance, liver fat, and oxidative stress [151].

Nobili et al. observed the distribution of intestinal *Bifidobacteria* and *Lactobacilli* in the feces of four groups: (1) children with obesity, (2) children with obesity and NAFLD, (3) children with obesity and NASH, and (4) healthy children. This study showed an increase in *Lactobacillus* spp. in NAFLD, NASH, and only obesity groups compared to healthy controls. In particular, the *L. mucosae* strain was significantly higher in the subjects with obesity, NAFLD, and NASH than in the healthy subjects. On the contrary, healthy controls had an abundance of *Bifidobacterium* spp., indicating their beneficial activity in liver disease [150].

The effectiveness of probiotic compound administration in improving weight and liver ultrasound is also confirmed by another randomized triple-blind study conducted in 64 children with obesity and with NAFLD [73]. In fact, Famouri et al. evaluated the administration of one capsule of probiotics for 12 weeks. The strains used were *Lactobacillus acidophilus* ATCC B3208, 3 × 10^9^ colony-forming units (CFU); *Bifidobacterium lactis* DSMZ 32269, 6 × 10^9^ CFU; *Bifidobacterium bifidum* ATCCSD6576, 2 × 10^9^ CFU; and *Lactobacillus rhamnosus* DSMZ 21690, 2 × 10^9^ CFU. The 64 patients (10–18 years) had a BMI > 85th percentile. They were equally divided into two groups: intervention and placebo. Although there were no significant changes in weight and body mass index, there was a significant decrease in mean ALT (from 32.8 ± 19.6 to 23.1 ± 9.9 U/L) and AST levels (from 32.2 ± 15.7 to 24.3 ± 7.7 U/L) and waist circumference (from 82.2 ± 14.7 to 80.3 ± 15.1) following the intervention. Finally, an improvement in liver ultrasound was also observed in both the intervention and placebo groups: it was normal in 17 (53.1%) subjects taking probiotics and in 5 (16.5%) of the placebo group [73].

An additional study conducted by Alisi et al. evaluated the beneficial effect of VSL#3 in children with obesity and steatohepatitis [152]. VSL#3 is the most extensively studied probiotic in NAFLD and is a mixture of eight probiotic strains (*Streptococcus thermophilus*, Bifidobacteria (*B. breve*, *B. infantis*, and *B. longum*), *Lactobacillus acidophilus*, *L. plantarum*, *L. paracasei*, and *L. delbrueckii* subsp. *bulgaricus*) [153]. The study was carried out on 44 (22 VSL#3 and 22 placebo) children with obesity and NAFLD. The primary endpoint was to assess liver health at 4 months by hepatic ultrasonography while the second one was to analyze whether changes had occurred in triglycerides, insulin resistance (assessed by HOMA index), ALT, BMI, and glucagon-like peptide 1 (GLP-1), and activated GLP-1 (aGLP-1). At 4 months of treatment with VSL#3, there was significant improvement in NAFLD in children, with decreased BMI and GLP-1 and increased aGLP1 [152].

Furthermore, the study by Goyal et al. evaluated the effects of VSL#3 in 116 children and adolescents (5–18 years old) with obesity and NAFLD. They divided them into four groups: (1) VSL#3 + lifestyle modification, (2) VSL#3, (3) lifestyle modification, and (4) placebo for 4 months. They assessed anthropometrical parameters (BMI, WC, and triceps skinfold thickness) and also biochemical parameters to evaluate lipid profile, fasting glycemia, high-sensitivity C-reactive protein, uric acid, and obesity hormones. The results showed that group 1 had better results both on anthropometric and biochemical parameters compared to probiotic and lifestyle modification alone [154].

Rodrigo et al. conducted a double-blind randomized placebo-controlled trial to evaluate the effects of probiotics on metabolic condition in 84 children (5–15 years old) with obesity and NAFDL. They were randomized either into a probiotic (*n* = 43) or a placebo (*n* = 41) group. Both groups received a structured diet and physical activity advice. After 6 months, BMI decreased in both groups [155]. Strangely, the probiotic group showed significant reductions in AST, ALT, AST/ALT ratio, and alkaline phosphatase compared to baseline, and these changes were not observed in the treated group. Metabolic parameters (lipid profile, glycemia, and insulinemia) did not significantly change in either group. Thus, probiotics showed no effectiveness over lifestyle modification in children with obesity and NAFLD [155].

Table 6 shows all included studies on probiotic supplementation in children with obesity and NAFLD.

## 6. Conclusions

Although NAFLD is one of the most common pediatric diseases and is now considered the hepatic manifestation of metabolic syndrome, a lack of recent clinical trials is present in the literature [23]. Interventions based on lifestyle modification through the adoption of proper eating habits and increased physical activity are the first-line treatments in the management of steatosis [21,55,93,156].

However, to date, it has not been defined which type of dietary treatment is most suitable in coping with this condition. There is a consensus in the literature that both the low-free-sugar diet and the Mediterranean diet are beneficial [99,100]. In particular, the latter has proved effective in counteracting the inflammatory state typical of metabolic syndrome due to its richness in polyunsaturated fats, polyphenols, vitamins, and carotenoids [110]. Additionally, the high presence of dietary fibers promotes a healthy gut microbiota, which in several studies seems to be inversely related to the development and progression of NAFLD [73,74].

In addition, the use of vitamin E, vitamin D, omega-3, and probiotics represents a good option to counteract NAFLD, blocking the development of fibrosis and the progression of liver disease [110,157]. Indeed, these molecules induce significant improvements in metabolic parameters such as a reduction in oxidative stress, insulin resistance, visceral and liver fat, and transaminases levels [110].

As there is a lack of trials in the pediatric population, further studies are needed to deeply evaluate which nutritional treatment and supplements are the most suitable in the management of NAFLD in children.

## Figures and Tables

**Table 1:** Percentiles classification according to WHO references [10].

0–2 Years (Children up to 24 Month) (WHO 2006)	2–5 Years (WHO 2006)	5–18 Years (WHO 2007)
Overweight risk > 85° percentile		
Overweight > 97° percentile	Overweight > 85° percentile	Overweight > 85° percentile
Obesity > 99° percentile	Obesity > 97° percentile	Obesity > 97° percentile

**Table 2:** Vitamin E supplementation in children with obesity and NAFLD.

References	Population (Age)	Duration	Treatment	Results
Ghergherehchi et al. [121]	33 children with obesity and NAFDL	6 months	Group 1: lifestyle modification + vitamin E (400 IU) Group 2: a lifestyle modification + placebo	Reduction in BMI, ALT, AST, triglycerides, total and LDL cholesterol equally in both groups except for LDL cholesterol that decreased more in group 1.
D’Adamo et al. [122]	42 prepubertal children with obesity and NAFLD	6 months	Group 1: vitamin E supplementation (600 mg/day) intervention group + nutritional and physical activity recommendations Group 2: control group + nutritional and physical activity recommendations	Reduction in BMI, waist circumference, and fasting glucose in both groups.
Shiasi et al. [124]	119 children with obesity and NAFLD	2 months	Group 1: vitamin E supplementation (800 IU/day or 400 IU/day) Group 2: metformin	No significant differences between the two treatments on liver ultrasonography.
Nobili [44]	80 children and pre-adolescents with biopsy-proven NAFLD	4 months	Treatment arm: 40 subjects received 7.5 mg of HXT and 10 mg of Vitamin E Placebo arm: the others received 240 mg medium- chain triglycerides (MCTs) as placebo	In the treatment arm, reduction in triglycerides and insulin levels, and an improvement in HOMA-IR were observed and also an increase in GSH and GSSG. The number of children affected by severe steatosis decreased.
Mosca [126]	70 children and pre-adolescents with biopsy-proven NAFLD	4 months	Treatment arm: 40 subjects Received 7.5 mg of HXT and 10 mg of Vitamin E Placebo arm: the others received 240 mg medium-chain triglycerides (MCTs) as placebo	IL-1β and TNF-α decreased in both groups, while IL-6 decreased and IL-10 increased significantly only in the HXT+VitE group.
Cho [129]	20 children aged 7–14 years with obesity and NAFLD	observation period: 16.76 ± 10.05 months of treatment 11.00 ± 9.40	First-line intervention: lifestyle modification Second-line intervention: vitamin E (800 IU/day) + UDCA (5–10 mg/day)	Vitamin E+ UDCA with BMI reduction determined a statistically significant improvements in AST, ALT, AST/ALT ratio, alkaline phosphatase, total bilirubin levels, and γ-GT at follow up, compared to the baseline.

**Table 3:** Vitamin D supplementation in children with obesity and NAFLD.

Reference	Population	Duration	Treatment	Results
El Amrousy et al., 2021 [131]	100 children with biopsy-proven NAFLD	6 months	Group 1: treatment group (2000 IU/day of vitamin D) Group 2: placebo group	Improvements in hepatic steatosis and lobular inflammation by liver biopsy and a decrease in AST, ALT, triglycerides, LDL, fasting insulin, and glycemia and HOMA-IR were observed. Moreover, vitamin D levels and HDL cholesterol significantly increased in the treated group.

**Table 4:** Omega-3 supplementation in children with obesity and NAFLD.

References	Population	Duration	Treatment	Results
Pacifico et al., 2015 [136]	51 children with obesity and NAFLD	6 months	Group 1 (*n* = 25): DHA supplementation Group 2 (*n* = 26): placebo	Reduction of 53.4% of liver fat in the DHA group compared to 22.6% in the placebo group. VAT and EAT reduced by 7.8% and 14.2%, respectively, in the DHA group, compared to 2.2% and 1.7% in the placebo group. Fasting insulin and triglycerides also decreased significantly in the DHA-treated group.
Janczyk et al., 2013 [137]	76 children with obesity and NAFLD	6 months	Omega-3 group: 450–1300 mg omega-3 fatty acids (containing DHA and EPA in 3:2 proportion) in two doses per day. Placebo group: the same dose of sunflower oil Both groups were invited to follow a hypocaloric diet and physical activity	ALT and GGT significantly decreased but no changes were observed in ALT levels compared to baseline.
Nobili et al., 2013 [138]	60 children with obesity and NAFLD	12 months	Group 1: DHA supplementation (250 mg/day and 500 mg/day Group 2: placebo	Reduction in both triglycerides and ALT levels and improvement in steatosis in group 1.
Spahis et al., 2015 [139]	21 children with obesity and NAFLD randomized in two groups and 33 healthy children	6 months	The study consisted of two phases: (1) Omega 3 supplementation (2 g of fish oil/daily providing 1.2 g of omega 3) to a group and a placebo (500 mg of sunflower oil with 3.75 U vitamin E.) to the other group for 3 months; (2) Both groups received the omega 3 supplementation, 4 capsules containing 500 mg of fish oil (300 mg of omega 3 with 3.75 U vitamin E) for the last 3 months.	The 21 NAFLD children showed an increase in the proportion of EPA and DHA at the expense of *n*-6 FAs after the treatment.
Spahis et al., 2018 [45]	20 male children with obesity and NAFLD	6 months	Participants were classified as sNAFLD (severe) or mNAFLD (mild). To sNAFLD patients, 2 g of *n*-3 PUFA was administered	Increased EPA and DHA concentrations in red blood cells and a reduction in FLI, ALT, and ALT/AST ratio and in lipid profile in the treated group.
Boyraz et al., 2015 [140]	108 children with obesity and NAFLD	12 months	PUFA group: diet + 1000 mg PUFA Placebo group: diet + placebo	Higher HDL levels and lower Triglycerides, insulin, glycemia, and HOMA-IR in the PUFA group.

**Table 5:** PUFA and vitamin D supplementation in children with obesity and NAFLD.

Reference	Population	Duration	Treatment	Results
Della Corte et al. [142]	41 children with obesity and NAFLD	24 weeks	Treatment arm: a mixture of vitamin D (800 IU) + DHA (500 mg) once/daily Placebo arm	Improved insulin resistance, lipid profile, ALT, and liver damage (NAFLD activity score).

**Table 6:** Probiotic supplementation in children with obesity and NAFLD.

References	Population	Duration	Treatment	Results
Nobili et al., 2018 [150]	115 = 61 children with obesity and NAFLD/NASH and 54 healthy controls	Observational study	4 groups: (1) children with obesity, (2) children with obesity and NAFLD, (3) children with obesity and NASH, and (4) healthy children.	Increased *Lactobacillus* spp. (especially *L. mucosae*) in groups 1, 2, and 3 compared to healthy controls.
Famouri et al., 2017 [73]	64 children with obesity and NAFLD	12 weeks	Group 1: 1 capsule of probiotic Group 2: placebo	Reduction in AST, ALT, and WC in group 1 and reduction in liver ultrasound in both groups.
Alisi et al., 2014 [152]	44 children with obesity and NAFLD	4 months	Group 1: VSL#3 (*n* = 22) Group 2: placebo (*n* = 22)	VSL#3 determined a significant improvement in NAFLD, with a decrease in BMI and GLP-1 and an increase in aGLP1.
Goyal et al., 2019 [154]	116 children and adolescents with obesity and NAFLD	4 months	4 groups: (1) VSL#3 + lifestyle modification, (2) VSL#3, (3) lifestyle modification, and (4) placebo	Group 1 showed a significant reduction in anthropometric and biochemical parameters compared to the other groups.
Rodrigo et al., 2021 [155]	84 children with obesity and NAFLD	6 months	Group 1: structured diet, physical activity, and probiotic Group 2: structured diet, physical activity, and placebo	BMI decreased in both groups; AST, ALT, AST/ALT ratio, and alkaline phosphatase decreased in the placebo group.

## Data Availability

Not applicable.
